# Mechanistic effects of percutaneous needle fasciotomy combined with stretching on early-stage skeletal muscle fibrosis in rats: focus on the TGF-β1/Smad signaling pathway

**DOI:** 10.1186/s13018-025-06400-z

**Published:** 2025-11-07

**Authors:** Han Zhang, Wanrong Li, Hanyu Zhang, MeiXuan Lu, Xinren Zhao, Jiahui Li, Rui Hu, Yi Zhang

**Affiliations:** 1 Acupotomy Research Institute, School of Acupuncture-Moxibustion and Tuina, Beijing University of Chinese Medicine, Beijing, 100029 China; 2 China-Japan Friendship Clinical Medical College, Beijing University of Chinese Medicine, Beijing, China; 3 Department of Anesthesiology, Beijing Tongren Hospital, Capital Medical University, Beijing, China; 4 School of Traditional Chinese Medicine, Beijing University of Chinese Medicine, Beijing, China; 5 The Second People’s Hospital of Kunming, Kunming, Yunnan Province China

**Keywords:** Skeletal muscle, Fibrosis, Percutaneous needle fasciotomy, TGF-β1/Smad

## Abstract

**Background:**

To investigate the effects of combination combining Percutaneous Needle Fasciotomy (PNF) with stretching(Str) on early-stage skeletal muscle fibrosis in rats, focusing on the transforming growth factor beta-1 (TGF-β1)/Smad signaling pathway.

**Methods:**

Twenty-four male Wistar rats were randomly divided into Control, immobilization(imm), Acupuncture(Apu) + Str, and PNF + Str groups (n = 6 each). After establishing a gastrocnemius fibrosis model by 4-week ankle imm in the right hindlimb ankle, intervention groups received either PNF (0.35 × 25 mm needle insertion with fascial release) or Apu (following an identical protocol), both combined with daily passive Str (3-N dorsiflexion for 7 days). Post-intervention assessments included functional tests (ankle joint range of motion, hindlimb grip strength, andgastrocnemius twitch/tetanic contractile forces), histological evaluation (collagen content via Masson's trichrome staining and immunofluorescence), and molecular analysis of TGF-β1/Smad pathway components**.**

**Results:**

Compared to the imm group, the PNF + Str group exhibited significantly better muscle performance, reduced histopathological damage, and favorable modulation of TGF-β1/Smad signaling markers. Importantly, these improvements were more pronounced than those in the Apu + Str group, wich demonstrates PNF's superior therapeutic effects in enhancing functional recovery, reducing fibrosis, and regulating the TGF-β1/Smad pathway.

**Conclusion:**

Our findings from this animal model demonstrate that PNF was superior to Apu in alleviating early-stage skeletal muscle fibrosis over the short term, potentially via modulation of the TGF-β1/Smad signaling pathway, though long-term effects warrant further investigation.

## Introduction

Skeletal muscle is essential for locomotion, postural support, and bodily stability [1]. A common pathological alteration in this tissue is fibrosis, which is characterized by excessive deposition of extracellular matrix (ECM) components and the progressive replacement of muscle tissue with fibrous tissue, thereby impairing normal muscle function [2]. Clinically, this condition often manifests as contractures, muscle weakness, pain, and restricted mobility. As a hallmark of severe muscle injury ,it is primarily caused by prolonged joint imm, physical inactivity, or sustained muscle contraction. Non-surgical management, including muscle stretching, acupuncture, Qigong [3], and pulsed electromagnetic field (PEMF) [4] therapy, are designed to enhance muscle extensibility and ameliorate muscular fibrosis.

PNF is a minimally invasive treatment technique for fascial contracture diseases, such as palmar fascial contracture. Its core mechanism involves the use of a rigid, large-bore needle with a cutting bevel to puncture percutaneously and mechanically section the pathological fibrous cord [4–6]. This directly releases the contracture, delays the process of muscular fibrosis, and improves joint function. Clinical studies have demonstrated that PNF has definite efficacy for palmar fascial contracture, and the long-term application of low-load tension postoperatively (achieved through Str or splinting) is a critical component for consolidating therapeutic outcomes [7–9]. This mechanical intervention helps control scar tissue hyperplasia, prevents the recurrence of joint contracture, and improves long-term hand function by promoting the organized remodeling of collagen.

Compared with open fasciectomy, PNF is widely regarded as an effective treatment option due to its simple procedure, feasibility under local anesthesia in an outpatient setting, low complications rate, and short recovery period [10]. It is important to clarify that PNF differs from Dry Needling in terms of treatment objective and mechanism: PNF is considered a surgical procedure that aims to achieve structural release of pathological anatomical tissue, while dry needling uses thin, flexible, solid Apu needles [11] to stimulate myofascial trigger points, eliciting localized and widespread effects.

Currently, the conventional PNF procedure predominantly utilizes a beveled injection needle. However, during puncture, the needle body is prone to deflection due to asymmetric force distribution, posing issues of suboptimal control and potential tissue damage [5]. Studies comparing needle tip shapes indicate that blunt-tip needles generate the highest puncture force [12]. The flat-blade needle knife employed in this study exhibits a relatively blunt characteristic at its tip compared to a beveled injection needle. Although this design increases the puncture force required, it affords superior straight-line advancement precision and depth control. The objective is to optimize the clinical efficacy of PNF by enabling safer and more thorough fascial sectioning. Furthermore, PNF is effective in ameliorating skeletal muscle fibrosis [13]; however, its underlying mechanisms remain unclear.

The TGF-β1/Smad signaling pathway is a master regulator of fibrogenesis in multiple organ systems [14]. It plays a pivotal role in fibrotic pathogenesis across multiple tissue types. Elevated TGF-β1 expression promotes myoblast differentiation and collagen synthesis, thereby promoting skeletal muscle fibrosis. For example, in tendon tissue,Yu et al. reported that engineering tendon stem cells to overexpress TGF-β1 directly led to enhanced fibrogenic differentiation and collagen deposition through the Smad2-dependent pathway, thereby promoting tendon fibrosis [15]. Previous studies have demonstrated that the combination of PNF and Str can ameliorate imm-induced gastrocnemius fibrosis through modulation of the Wnt/β-catenin signaling pathway [13, 16]. Studies have also indicated that the Wnt/β-catenin pathway activation upregulates TGF-β1 expression [17], which subsequently activates the TGF-β1/Smad signaling cascade to collectively orchestrate the progression of skeletal muscle fibrosis.

This study aimed to investigate the therapeutic effects of the combination of PNF and Str on skeletal muscle fibrosis and to elucidate its potential association with TGF-β1/Smad signaling pathway modulation. Our preliminary studies demonstrated the superior efficacy of PNF-Str combination therapy over Str alone in attenuating gastrocnemius muscle fibrosis. The present study specifically compares the therapeutic outcomes of different needle devices to optimize intervention protocols.

## Methods

### Animal grouping and modeling

Twenty-four healthy male Wistar rats (8 weeks, 300 ± 20 g, specific pathogen-free (SPF)grade) were procured from Beijing Vital River Laboratory Animal Technology Co., Ltd. (Beijing, China; License No.SCXY (Jing) 2016–0006) . The animals were housed in the Experimental Animal Center of Beijing University of Chinese Medicine under controlled conditions (20–25 °C, 50%-60% humidity, 12-hour light/dark cycle) with ad libitum access to food and water. The study protocol was approved by the Animal Ethics Review Committee of Beijing University of Chinese Medicine (Approval No. BUCM-4-2023051802-2216), and all experimental procedures strictly complied with the institutional guidelines for laboratory animal welfare. To minimize bias, all personnel involved in data collection and evaluation were blinded to the group allocation throughout the behavioral, histological, and molecular analyses. After a 7-day acclimatization period, the 24 rats were randomly allocated into the following groups (n = 6/group): Control,Immobilization(imm), Acupuncture + Str (imm + Apu + Str), and Percutaneous Needle Fasciotomy + Str (imm + PNF + Str). All rats except those in the Control group were immobilized in plantarflexion position for 4 weeks using the rat fixation device developed by Coutinho et al. [13, 18]. 

 Researchers daily examined the affected limbs for ulceration and edema. Successful modeling was confirmed by the following criteria [16]: a significant reduction in the range of motion (ROM), shortened stride length with a limping gait, and markedly decreased movement distance and duration.

### Interventions

**Fig. 1:**
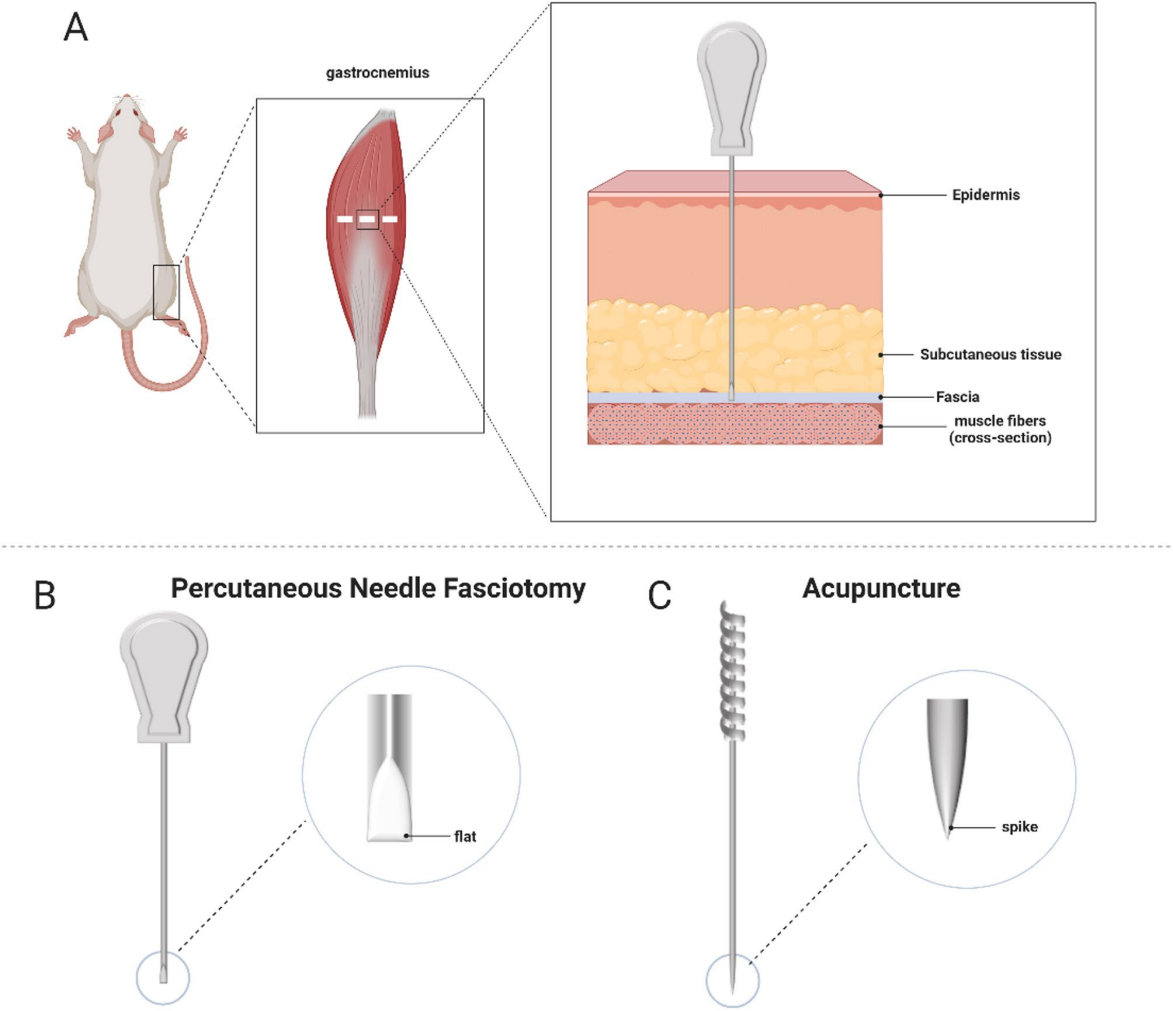
Acupuncture Points and Needle Tool Comparison: PNF Needles versus Apu Needles. **A** PNF intervention on the gastrocnemius muscle; **B** PNF needle (0.35 mm × 25 mm); **C** Apu needle (0.35 mm × 25 mm)

Rats in the Control and imm groups received standard handling and restraint without any therapeutic intervention. In the imm + PNF + Str group, a 0.35 mm × 25 mm PNF needle (Wujiang Yunlong Medical Devices Co. Ltd, Jiangsu, China; Fig. 1B) was inserted perpendicular to the muscle fiber orientation at three equidistant points along the midbelly of the right gastrocnemius muscle. The needle tip penetrated 0.3–0.5 cm through the superficial fascia layer, as confirmed by a distinct loss-of-resistance sensation, and care was taken to avoid deeper muscle penetration . Each rats in this group received only a single session of PNF (Fig. 1A).

After the PNF procedure, a mechanical Str protocol was immediately initiated and sustained for 7 consecutive days. This protocol was administered once daily and consisted of 10 stretches to the right ankle joint. A dorsiflexion force of 3 N—determined from pilot studies to be a submaximal, tolerable level that effectively stretches the gastrocnemius without causing injury—was applied perpendicular to the plantar surface using a push–pull gauge. Each stretch was held for 30 s, followed by a 30-s rest period.

In the imm + Apu + Str group, rats received acupuncture with a 0.35 mm × 25 mm Apu needle (Fig. 1C) ,with needle-insertion points, depth, and Str method identical to those in the imm + PNF + Str group.

After completion of the interventions, rats fromall four groups underwent assessment for joint ROM and hindlimb grip strength. The animals were then anesthetized by intraperitoneal injection of 20% urethane (0.6 mL/100 g body weight). Subsequently, the gastrocnemius muscles were exposed for in situ contractility testing. Upon completion of the functional measurements, the rats were euthanized by anesthetic overdose; all procedures were performed in accordance with institutional animal care guidelines. The right hindlimb gastrocnemius muscle was then completely dissected and collected for further analyses.

For each muscle sample, following continuous sectioning, at least three non-consecutive sections (spaced at least 50 μm apart) were selected for analysis. From each section, four non-overlapping fields of view were randomly captured in the central region of the muscle belly. Areas with inherently high collagen density, such as the myotendinous junction and myofascial edges, were excluded to ensure quantitative consistency. The final data for each sample represented the average of measurements from at least 12 randomly selected fields, thereby ensuring representativeness and statistical power in the quantitative assessment.

### Muscle-performance testing

The hindlimb ankle dorsiflexion ROM [19] was defined as the maximal dorsiflexion angle (0°–160°) formed between two reference lines: one parallel to the longitudinal axis of the fibula and the other parallel to the plantar surface of the heel.

Previous studies [13, 20] have shown that a minimum force of 0.3 N is required to achieve passive dorsiflexion in rats. Accordingly, rats were positioned in left lateral recumbency, and a push–pull dynamometer (Dongguan Donglaida Electronics Co., Ltd, Dongguan, China) was used to apply a 0.3 N force perpendicular to the right plantar surface to induce maximal ankle dorsiflexion. The ROM was measured three times using a digital angle ruler (Dongguan Sanliang Precision Instruments Co., Ltd, Dongguan, China), and the average value was recorded.

Hindlimb grip strength is an important indicator of hindlimb muscle strength [21]. The rat's hindlimbs were placed on a grid plateof a grip strength meter (BIOSEB, USA). When the hindlimbs were observed to grip the crossbar, the tail was pulled horizontally backward with gradually increasing force until the hindlimbs released the crossbar. The reading on the instrument at this point represented the rat's maximum grip strength, which was measured three times and averaged.

Upon completion of the interventions, the rats were anesthetized, and their gastrocnemius muscles were carefully dissected. Then, the distal tendon was securely ligated with suture thread, and the muscle's insertion point was surgically transected. The suture was then attached to an FT-100S force transducer (Chengdu Taimeng Software Co., Ltd, Chengdu, China), which was firmly mounted on a metal stand to maintain the gastrocnemius muscle at its resting length while ensuring the connecting line remained perfectly vertical to the work surface. The transducer's output was connected to a BL-420S biological signal acquisition system (TM001-1702-01-080-002; Chengdu Taimeng Software Co., Ltd, Chengdu, China) for data recording and analysis.

The output terminals (positive and negative) of the stimulating electrodes were directly connected to both ends of the gastrocnemius muscle. Single stimuli were initially applied with the following parameters: pulse width, 1 ms; delay, 1 s. The stimulus intensity was gradually increased from zero in 0.1-V increments until maximal twitch tension was achieved, at which point both the maximal twitch force (N) and optimal stimulus intensity were recorded. Subsequently, at the optimal stimulus intensity, the frequency was progressively increased until complete tetanic contraction was observed, and the maximal tetanic force (N) was recorded. Throughout the measurements, Krebs–Henseleit solution (Procell, Wuhan, China) was continuously applied to the muscle to maintain physiological conditions. A 30 s rest period was maintained between stimulations to limit the influence of muscle fatigue on the results.

### Weight and length of muscle

Upon completion of the muscle contractility measurements, the right hindlimb gastrocnemius muscles were promptly excised. Before measurement of gastrocnemius muscle weight, the muscle samples were gently blotted to remove excess surface liquid while avoiding excessive compression or tissue damage. The measurements were obtained using an electronic balance (accuracy: 0.1 mg), and the muscle samples were handled with forceps or other clean instruments to prevent direct hand contact and avoid contamination of the measurement results. Before measurement of gastrocnemius muscle length, the muscle was gently extended from its origin (medial femoral condyle) to its insertion (Achilles tendon attachment) to achieve a natural, non-tensioned state, avoiding either overstretching or slackness. The linear distance between these anatomical landmarks was then measured using a vernier caliper (accuracy: 0.02 mm) and recorded as the muscle length.

### Masson staining

After standard dehydration and paraffin embedding, 4-μm-thick transverse sections of the gastrocnemius muscle were obtained. For deparaffinization and rehydration, the sections were sequentially processed through xylene I for 20 min, xylene II for 20 min, absolute ethanol I for 5 min, absolute ethanol II for 5 min, and 75% ethanol for 5 min, followed by rinsing under running tap water. Using a Masson staining kit (Beijing Zhongke Wanbang Biotechnology Co., Ltd, Beijing, China), sections were immersed in Masson A solution overnight, then rinsed with tap water. The sections were then immersed in a 1:1 mixture of Masson B and C solutions for 1 min, rinsed with tap water, differentiated with 1% acid alcohol, and rinsed again. The sections were then stained in Masson D solution for 6 min, followed by rinsing in tap water. Next, the sections were immersed in Masson E solution for 1 min. Without water rinsing, excess liquid was briefly drained, and the sections were directly transferred to Masson F solution for 2–30 s. The sections were differentiated with 1% glacial acetic acid, and then dehydrated through two changes of absolute ethanol. After the third absolute ethanol bath for 5 min, the sections were cleaned in xylene for 5 min and then mounted with neutral balsam. Microscopic examination and photography of gastrocnemius cross-sections were performed, andthe cross-sectional area (CSA) and collagen volume fraction (CVF%) were measured using ImageJ software.

### Immunofluorescence

Tissue samples were processed using a cryostat (CM1950, Germany) to obtain 10-µm-thick sections mounted on adhesive microscope slides (Citotest, Jiangsu, China) for immunofluorescence staining. The sections were air-dried at room temperature and washed three times with TBST (5 min each). Triton X-100 (Coolaber, Beijing, China) was applied for 20 min at room temperature to enhance membrane permeability, followed by three TBST washes (5 min each). Nonspecific binding sites were blocked with goat serum (Bioss, Beijing,China) for 1 h before incubation with the primary antibody overnight at 4 °C. After three TBST washes (5 min each), the sections were incubated with secondary antibodies for 2 h at room temperature and washed again three times with TBST (5 min each). Mounting Medium, antifading (with DAPI) (Solarbio, Beijing, China) was applied before coverslipping. Stained gastrocnemius sections were examined under a fluorescence microscope at 20x magnification, and CoraLite 488 and 594 fluorescence intensities were quantified using ImageJ. Antibodies against collagen I (1:100, Abcam, UK) and collagen III (1:500, Proteintech, Wuhan, China) were used as the primary antibodies. The secondary antibodies were Goat Anti-Rabbit IgG(h + l) CoraLite 488 (1:5000, Proteintech, Wuhan, China) and Goat Anti-Rabbit IgG(h + l) CoraLite 594 (1:500, Proteintech, Wuhan, China).

### Real-time PCR

After specimen collection, rats were euthanized by an anesthetic overdose. The muscle samples were then divided for processing: one portion was immediately frozen in liquid nitrogen and stored at − 80 °C, while another portion was fixed in Carnoy's solution (zkwb-bio, Beijing, China) for 24 h for subsequent analyses.

Total RNA was homogenized and extracted using RNA isolation reagent (Thermo, USA). The extracted RNA was reverse-transcribed into complementary DNA (cDNA) in accordance with the instructions of the reverse transcription kit. Real-time polymerase chain reaction (PCR) was performed using Power Up™ SYBR™ Green Master Mix (Thermo, USA) for cDNA amplification. Specific PCR primers for glyceraldehyde-3-phosphate dehydrogenase (GAPDH), TGF-β1, Smad2, Smad3, Smad7, interferon gamma (IFN-γ), and alpha smooth muscle actin (α-SMA) were designed on the basis of published sequences. The PCR conditions were set as follows: initial denaturation at 95 °C for 5 min, followed by 45 cycles of 95 °C for 30 s, 60 °C for 30 s, and 72 °C for 30 s. The relative expression levels of target genes were calculated using the 2^−ΔΔCT^ method and normalized to GAPDH mRNA expression. The forward and reverse primers are listed in Table 1.

**Table 1:** Primers used in this study

Gene	Primers sequences(5′–3′)	Length
TGF-β1	Forward: GACCGCAACAACGCAATCTATGAC	94
	Reverse: CTGGCACTGCTTCCCGAATGTC	94
Smad2	Forward: GTCGTCCATCTTGCCATTCACTC	95
	Reverse: GTTCTCCACCACCTGCTCCTC	95
Smad3	Forward: AGGGCTTTGAGGCTGTCTACC	102
	Reverse: TGCTGGTCACTGTCTGTCTCC	102
Smad7	Forward: AAGAGGCTGTGTTGCTGTGAATC	123
	Reverse: CGGGTATCTGGAGTAAGGAGGAG	123
IFN-γ	Forward: CAACCCACAGATCCAGCACAAAG	86
	Reverse: TCCGCTTCCTTAGGCTAGATTCTG	86
α-SMA	Forward: AGGGAGTGATGGTTGGAATGGG	110
	Reverse: GGTGATGATGCCGTGTTCTATCG	110
GAPDH	Forward: AAGTTCAACGGCACAGTCAAGG	123
	Reverse: GACATACTCAGCACCAGCATCAC	92

### Western blot analysis

Total protein was extracted from the gastrocnemius muscle using RIPA lysis buffer (Solarbio, Beijing, China) supplemented with a protease inhibitor cocktail (Solarbio, Beijing, China). Protein concentration was determined using a BCA assay kit (Solarbio, Beijing, China). The proteins were separated by sodium dodecyl sulfate (SDS)-polyacrylamide gel electrophoresis (PAGE). Separated proteins were electrophoretically transferred onto polyvinylidene difluoride (PVDF) membranes.

The membranes were blocked with rapid blocking buffer for 25 min at room temperature. After washing three times with Tris-buffered saline with Tween 20 (TBST; 10 min per wash), the membranes were sectioned and incubated with primary antibodies overnight at 4 °C. Next, the membranes were washed three times with TBST (10 min per wash), incubated with the secondary antibody for 1 h at room temperature, and washed again three times with TBST (10 min per wash). Protein bands were visualized using an enhanced ECL substrate and imaged with a chemiluminescence detection system. Relative protein expression was quantified using ImageJ software, normalized to the expression of GAPDH. Antibodies against TGF-β1 (1:500, bioss, Beijing, China), Smad2 (1:1000, bioss, Beijing, China), Smad3 (1:1000, bioss, Beijing, China), Smad7 (1:1000, bioss, China), IFN-γ (1:1000, bioss, Beijing, China), GAPDH (1:2000, bioss, Beijing, China), α-SMA (1:1000, bioss, Beijing, China) were used as the primary antibodies. The secondary antibody was Goat Anti-Rabbit IgG H&L (HRP-conjugated, 1:5000, bioss, Beijing, China).

### Statistical analysis

All experimental data were processed using GraphPad Prism 10 for visualization and SPSS 29.0 for statistical analysis. The rigorous validation included normality testing using the Kolmogorov–Smirnov test and assessment of the homogeneity of variance using Levene's test. Parametric data (normally distributed with homogeneous variance) were analyzed using one-way analysis of variance (ANOVA) followed by least significant difference (LSD) post-hoc tests for pairwise comparisons. Non-parametric data were analyzed using Kruskal–Wallis H tests. Statistical significance was set at *p* < 0.05 for all analyses.

## Results

### Muscle-performance testing

**Fig. 2:**
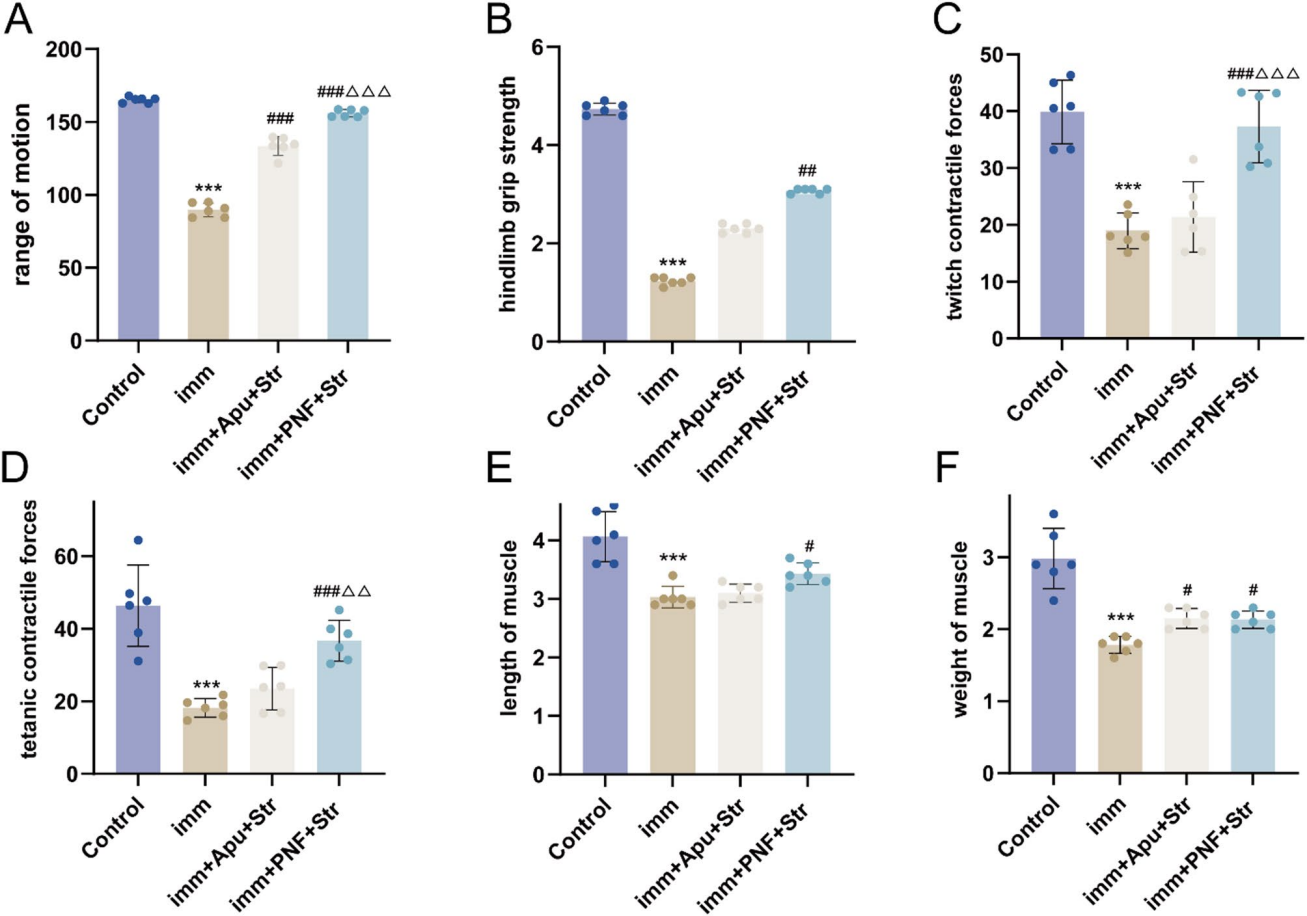
Effects of PNF on muscle-function parameters. **A** range of motion; **B** hindlimb grip strength; **C** twitch contractile forces; **D** tetanic contractile forces; **E** length of muscle; **F** weight of muscle. Statistical comparisons: ***P < 0.001 versus Control group; #*P* < 0.05, ##*P* < 0.01, ###*P* < 0.001 versus imm group; ΔΔ*P* < 0.01, ΔΔΔ*P* < 0.001 versus imm + Apu + Str group

In comparison with the Control group, the imm group showed significantly lower values in all functional parameters, including ROM, hindlimb grip strength, twitch contractile force and tetanic contractile forces (all *p* < 0.001). The imm + PNF + Str group showed statistically significant recovery of all functional parameters in comparison with the imm group (*P* < 0.01 to *P* < 0.001). In contrast, in comparison with the imm group, the imm + Apu + Str intervention only led to a significant improvement in ROM (*p* < 0.001), with no statistically significant benefits on muscle strength or contractility. Notably, the functional outcomes in the imm + PNF + Str group were significantly superior to those in the imm + Apu + Str group, particularly in ankle ROM, twitch contractile force, and tetanic contractile force (*P* < 0.01 to *P* < 0.001)(Figure 2A-D).

### Weight and length of gastrocnemius muscle

In comparison with the Control group, the imm group showed significant alterations in gastrocnemius muscle length and weight (all *p* < 0.001). The imm + PNF + Str group showed statistically significant recovery of both muscle length and weight in comparison with the imm group (*P* < 0.05). In contrast, the imm + Apu + Str intervention only led to a significant improvement in muscle weight (*p* < 0.05), with no statistically significant effect on muscle length. The restorative effect on muscle length in the imm + PNF + Str group was significantly superior to that in the imm + Apu + Str group (Fig. 2E, F).

### Masson staining

**Fig. 3:**
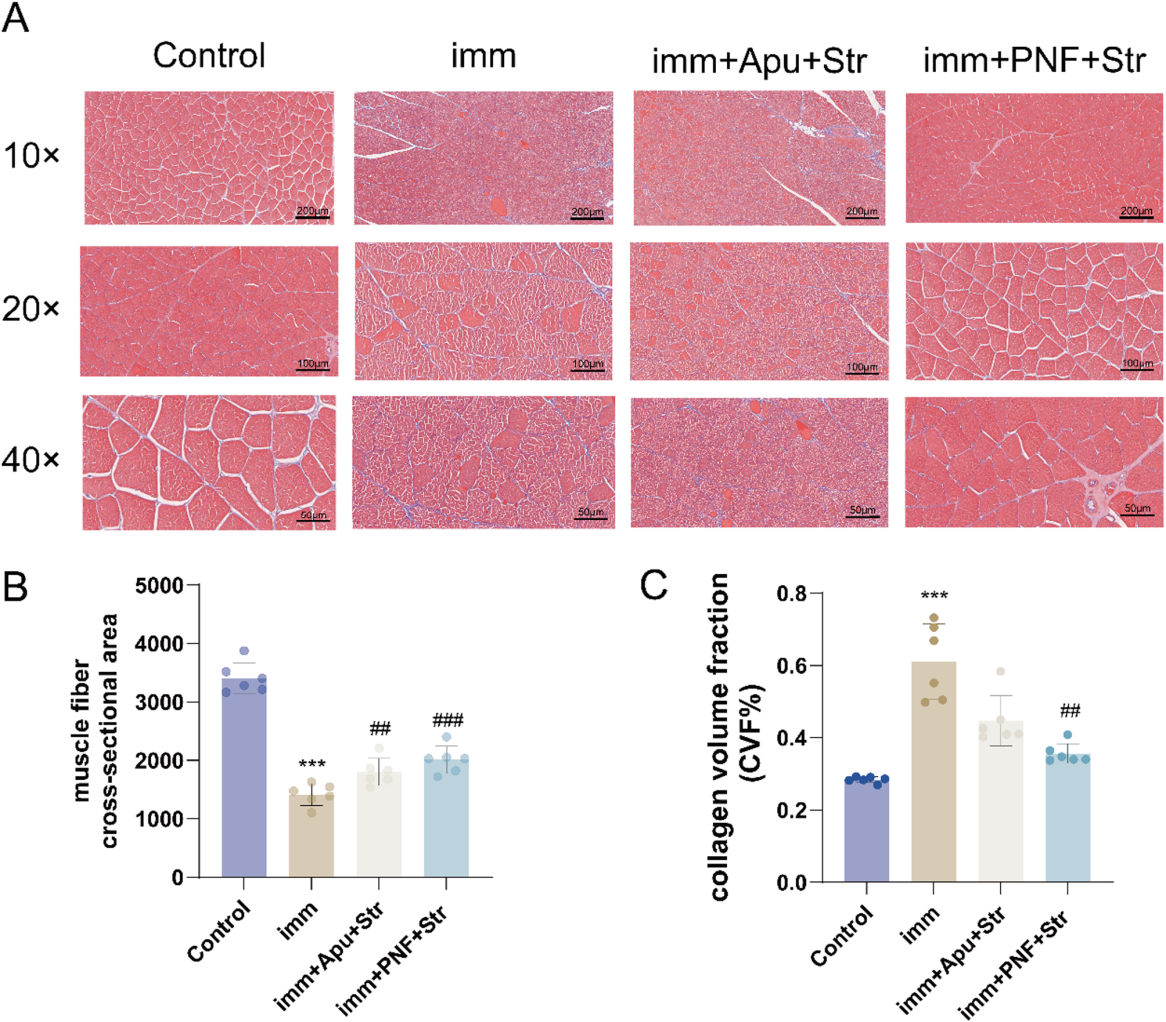
Effects of PNF on muscle fibrosis. **A** Transverse sections of gastrocnemius muscle stained with Masson's trichrome (magnification: 10×, 20×, 40×); **B** cross-sectional area (CSA) of gastrocnemius muscle; **C** collagen volume fraction (CVF%) of gastrocnemius muscle. Statistical comparisons:***P < 0.001 versus Control group; ###*P* < 0.001 versus imm group; Δ*P* < 0.05 versus imm + Apu + Str group

As shown in the figures, collagen fiber deposits were stained blue, muscle fibers were stained red, and nuclei appeared dark. In the Control group, muscle fibers were neatly arranged, while the Imm group exhibited a loose, fragmented, and disorganized gastrocnemius muscle structure with widened intermuscular spaces surrounded by abundant blue-stained collagen fibers. In comparison with the Imm group, both imm + PNF + Str and imm + Apu + Str groups showed reduced intermuscular spaces and more organized, thinner muscle fibers. Notably, the imm + PNF + Str group demonstrated better-aligned skeletal muscle fibers with fewer blue-stained collagen deposits (Fig. 3A).

The imm group significantly evidenced by a reduced muscle fiber cross-sectional area (CSA) and an increased collagen volume fraction (CVF%) compared to the Control group (all *p* < 0.001). Both the imm + PNF + Str and imm + Apu + Str groups significantly improved the CSA (*p* < 0.01, *p* < 0.001). However, only the imm + PNF + Str treatment resulted in a significant reduction of the CVF% compared to the imm group (*p* < 0.01), while the change in the imm + Apu + Str group was not statistically significant (Fig. 3B, C).

### Immunofluorescence

In this study, the mean fluorescence intensity of collagen I and III was evaluated using immunofluorescence staining. In comparison with the Control group, the Imm group exhibited significantly greater mean fluorescence intensity of collagen I (*P* < 0.001). Both intervention groups showed lower collagen I expression than the Imm group, with the imm + PNF + Str group demonstrating more pronounced reduction (*P* < 0.001) than the imm + Apu + Str group (*P* < 0.01).

**Fig. 4:**
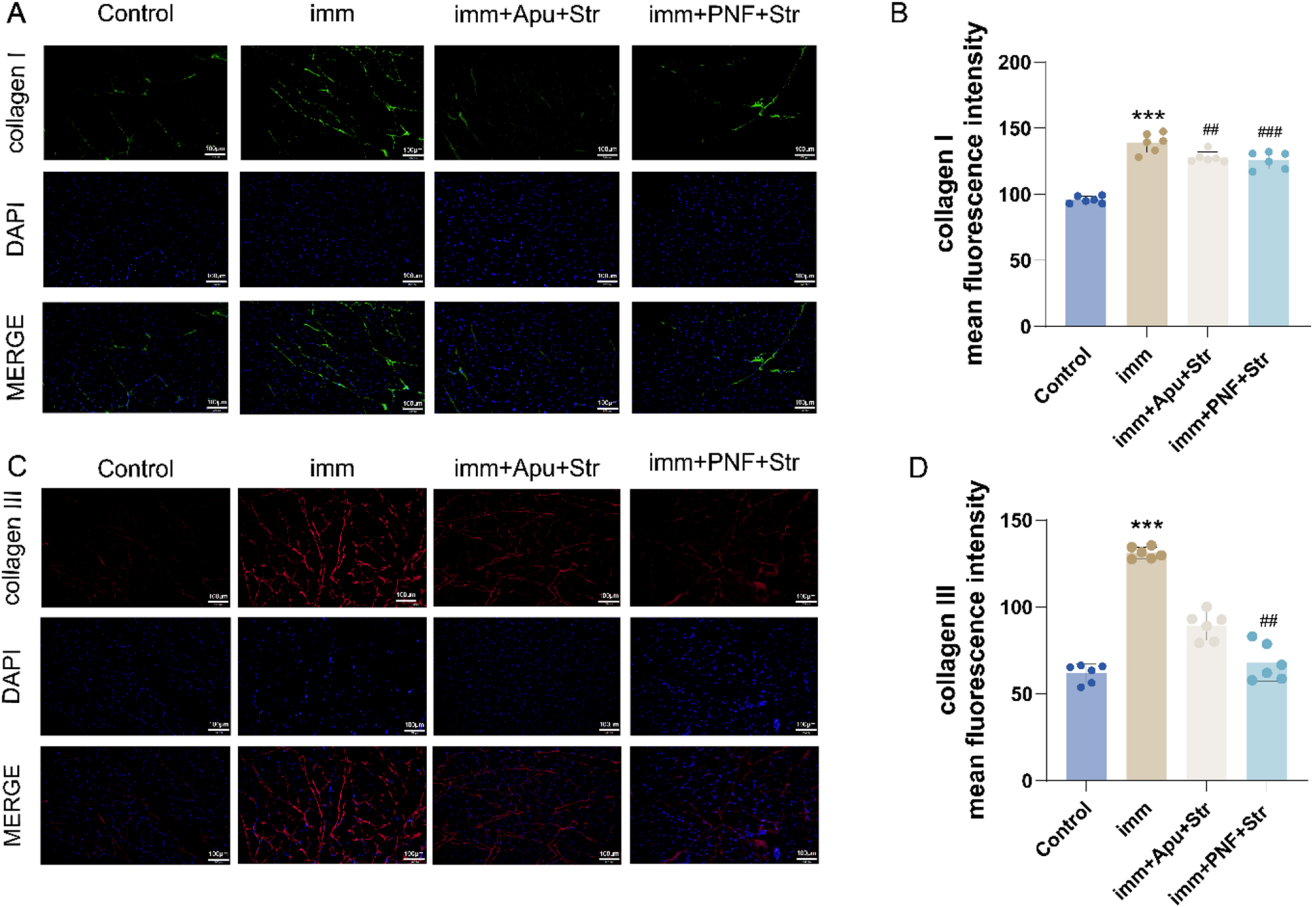
Effects of PNF on immunofluorescence intensity indicating collagen I and III expression. **A** Representative immunofluorescence images of collagen I (green), 20 × magnification; **B** quantitative analysis of collagen I mean fluorescence intensity; **C** representative immunofluorescence images of collagen III (red), 20 × magnification; **D** quantitative analysis of collagen III mean fluorescence intensity. Statistical comparisons: ***P < 0.001 versus Control group; ##*P* < 0.01, ###*P* < 0.001 versus imm group

Similarly, the Imm group showed significantly elevated collagen III intensity than the Control group (*P* < 0.001). While the imm + PNF + Str group showed a significant decrease in collagen III expression in comparison with the Imm group (*P* < 0.01), the reduction in the imm + Apu + Str group failed to reach statistical significance (Fig. 4B–D).

### Real-time PCR

**Fig. 5:**
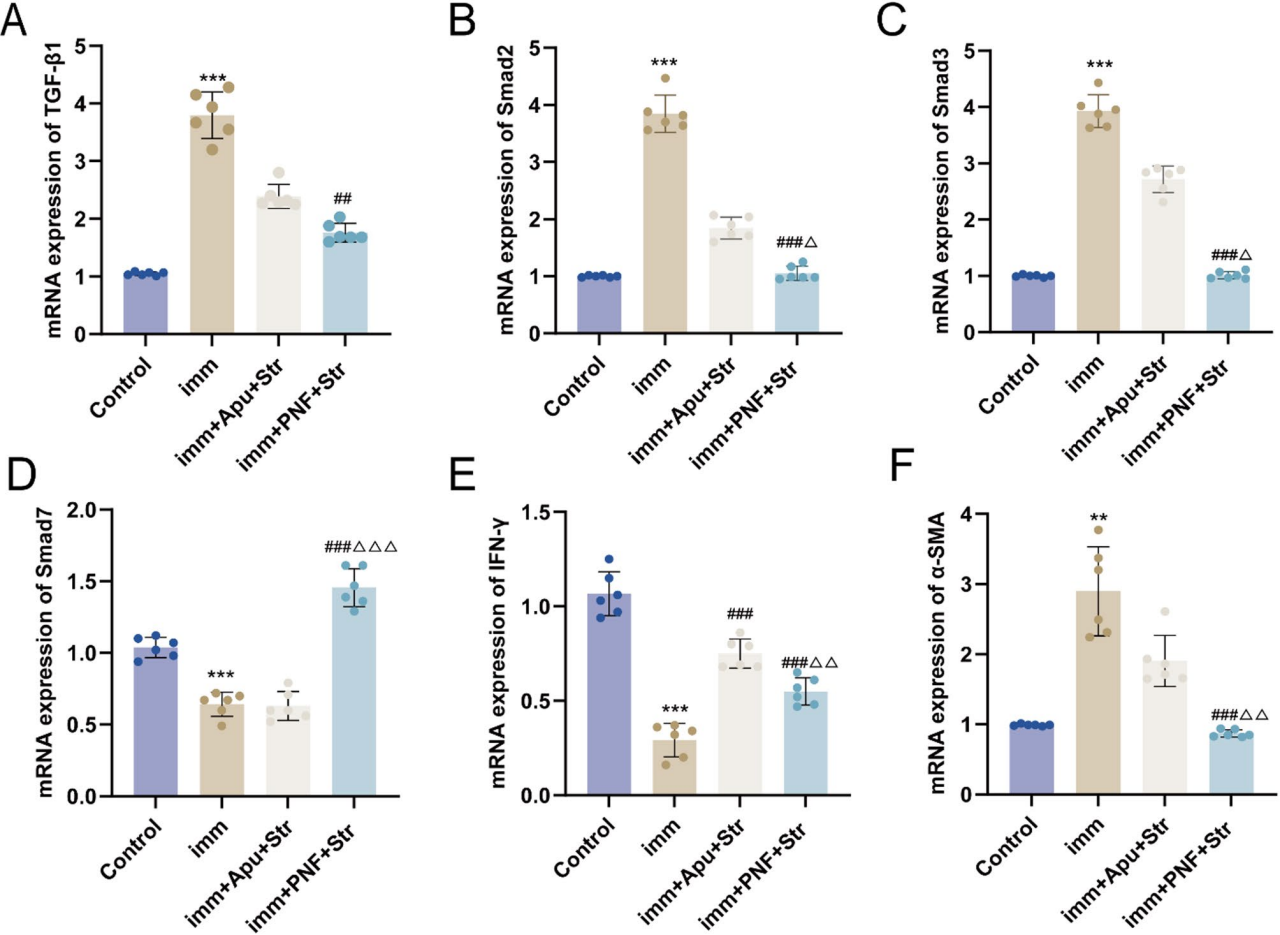
Real-time PCR analysis of gene expression. **A** TGF-β1; **B** Smad2; **C** Smad3; **D** α-SMA; **E** Smad7; **F** IFN-γ. Statistical comparisons: **P < 0.01, ***P < 0.001 versus Control group; ##*P* < 0.01, ###*P* < 0.001 versus imm group; Δ*P* < 0.05, ΔΔ*P* < 0.01, ΔΔΔ*P* < 0.001 versus imm + Apu + Str group

In comparison with the Control group, the Imm group showed significantly elevated mRNA levels of TGF-β1, Smad2, and Smad3 (all *P* < 0.001), along with increased α-SMA expression (*P* < 0.01). In comparison with the Imm group, the imm + PNF + Str group demonstrated significantly lower TGF-β1 expression (*P* < 0.01) and markedly lower Smad2, Smad3, and α-SMA expression (all *P* < 0.001). No significant changes were observed in the imm + Apu + Str group. In comparison with the imm + Apu + Str group, the imm + PNF + Str group showed no significant difference in TGF-β1 expression and lower expression levels of Smad2 (*P* < 0.05), Smad3 (*P* < 0.05), and α-SMA (*P* < 0.01) (Fig. 5A–C, E).

The Imm group exhibited significantly lower Smad7 and IFN-γ mRNA levels (both *P* < 0.001) than the Control group. Although imm + PNF + Str restored the levels of both markers (both *P* < 0.001), imm + Apu + Str only increased the IFN-γ level (*P* < 0.001). Notably, imm + PNF + Str outperformed imm + Apu + Str in upregulating Smad7 (*P* < 0.001) and IFN-γ (*P* < 0.01) expression (Fig. 5D, F).

### Western blot analysis

**Fig. 6:**
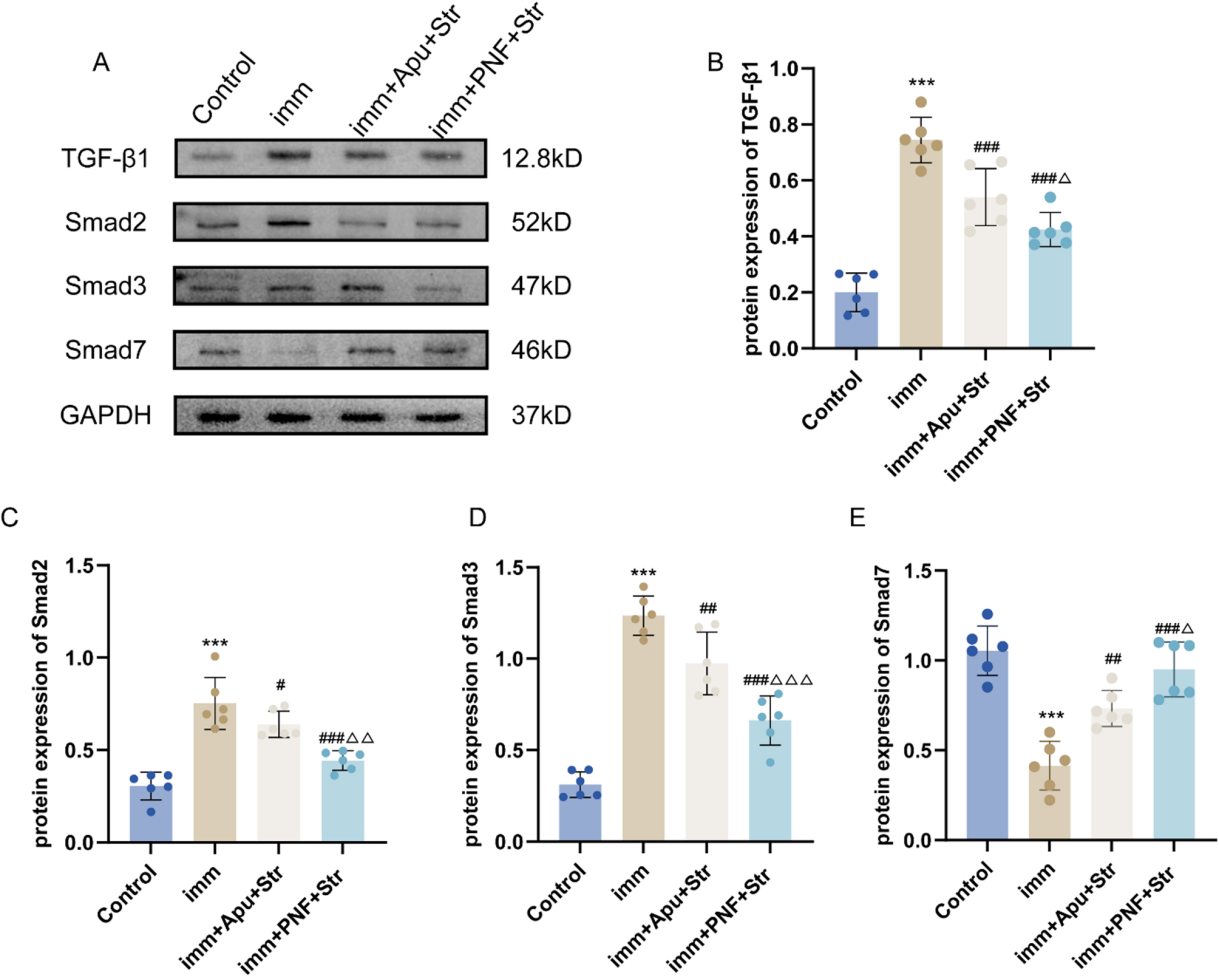
Western blot analysis of antibody protein expression. **A** Representative western blot bands; **B** TGF-β1; **C** Smad2; **D** Smad3; **E** Smad7. Statistical comparisons: ***P < 0.001 versus Control group; #*P* < 0.05, ##*P* < 0.01, ###*P* < 0.001 versus imm group; Δ*P* < 0.05, ΔΔ*P* < 0.01, ΔΔΔ*P* < 0.001 versus imm + Apu + Str group

**Fig. 7:**
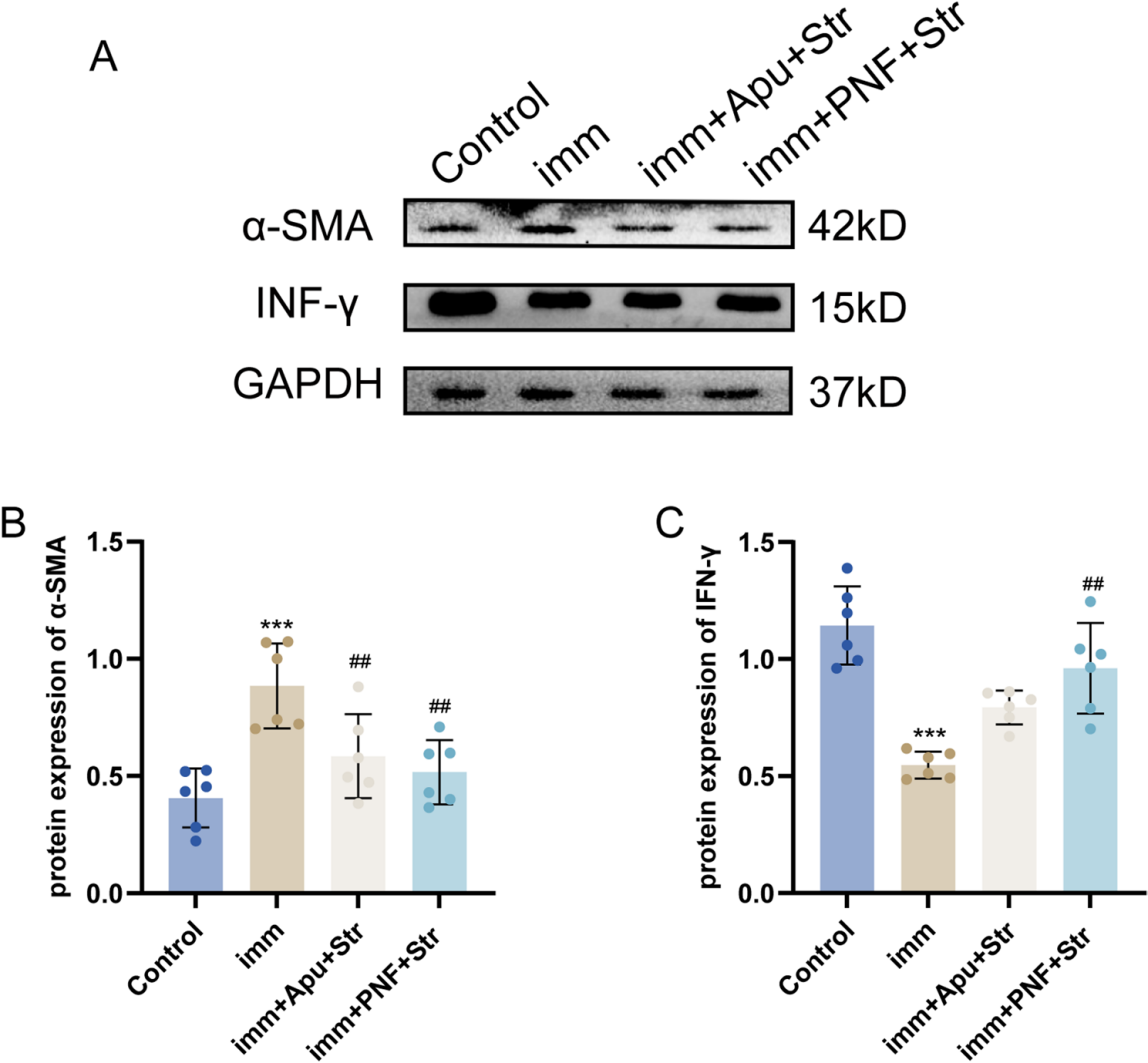
Western blot analysis of antibody protein expression. **A** Representative western blot bands; **B** α-SMA; (C) IFN-γ. Statistical comparisons: ***P < 0.001 versus Control group; ##*P* < 0.01 versus imm group

In this study, western blot analysis revealed significantly increased protein expression of TGF-β1, Smad2, Smad3, and α-SMA in the Imm group in comparison with the Control group (all *P* < 0.001). Relative to the Imm group, the imm + PNF + Str group showed significantly reduced expression of TGF-β1, Smad2, and Smad3 (all *P* < 0.001) along with decreased α-SMA expression (*P* < 0.01), while the imm + Apu + Str group exhibited significant reductions in TGF-β1 (*P* < 0.001), Smad2 (*P* < 0.05), Smad3 (*P* < 0.01), and α-SMA (*P* < 0.01) expression without significant changes in Smad7 expression. Notably, the imm + PNF + Str group demonstrated superior inhibitory effects than the imm + Apu + Str group for TGF-β1 (*P* < 0.05), Smad2 (*P* < 0.01), and Smad3 (*P* < 0.001) expression (Figs. 6C, D, 7B).

In comparison with the Control group, the Imm group showed significantly decreased protein expression of Smad7 and IFN-γ (all *P* < 0.001). Relative to the Imm group, the imm + PNF + Str group exhibited significantly increased expression of Smad7 (*P* < 0.001) and IFN-γ (*P* < 0.01), while the imm + Apu + Str group demonstrated significantly elevated Smad7 expression (*P* < 0.01). Furthermore, the imm + PNF + Str group showed significantly higher Smad7 protein expression than the imm + Apu + Str group (*P* < 0.05) (Figs.6E, 7C).

## Discussion

In this study, we successfully established a rat skeletal muscle fibrosis imm through 4-week imm, which effectively maintained the hindlimb muscles in a chronic disuse state. The consequent skeletal muscle fibrosis showed pathological characteristics closely resembling the pathogenesis of human skeletal muscle fibrosis. In comparison with the Control group, the Imm group exhibited significantly reduced joint ROM, hindlimb grip strength, gastrocnemius single contraction force and tetanic contraction force, and gastrocnemius muscle length and weight. Masson staining revealed a loose and fragmented gastrocnemius structure with enlarged intermuscular spaces and significantly increased collagen volume fraction. These findings demonstrate that 4-week imm successfully induced skeletal muscle fibrosis in rats, consistent with our previous reports [5].In this study, we established both imm + Apu + Str and imm + PNF + Str groups to compare the therapeutic effects of different needle instruments on skeletal muscle fibrosis. The rationale for performing Str after PNF or Apu intervention is related to the incisions created on the muscular surface fascia. The fascia provides stable mechanical support for muscles. Str following fascial incision effectively enhances muscle elongation and improves joint ROM, promoting normal activity of the injured muscles along with their synergistic and antagonistic muscles and thereby enhancing muscular function. The flat-blade tip used in PNF and the sharp needle tip in Apu create distinct wound morphologies, potentially resulting in differential elongation effects during subsequent Str interventions. According to the Chinese medicine literature "Acupuncture and Moxibustion Experience Prescription • Foot and Knee, "the hand and foot tendons are contracted and astringent. After stabbing the tendons four or five times with a round, sharp needle, then have assistants stretch the contracted areas, Str the contracted muscles until relief is achieved—remarkably effective.” This therapeutic method is highly consistent with the underlying principles of modern PNF in releasing tissue contracture, since round-sharp needles were used as the instruments at that time. Round-sharp needles featured thicker diameters that created larger puncture wounds and consequently produced more pronounced tissue-releasing effects. Ancient Apu needles had thicker diameters that enabled dual functionality of both acupoint stimulation and tissue release, whereas modern needles have reduced diameters that create smaller puncture wounds. Although the smaller wounds improve safety and patient acceptance, they diminish the tissue-releasing function to some degree. The flat-blade design of the PNF needle tip can be regarded as an innovative modification of traditional thick needles that is specifically engineered to enhance tissue-releasing functionality, thereby objectively preserving and intensifying the therapeutic releasing effects characteristic of ancient needle techniques. In the current experiment, the combination of PNF and Str demonstrated significantly superior improvements in multiple muscle-function parameters, including joint ROM, hindlimb grip strength, gastrocnemius twitch contraction force and tetanic contraction force, in comparison with the combination of Apu and Str. These findings suggest that variations in puncture wound size and configuration, as the foundational basis for Str interventions, may substantially influence therapeutic outcomes.

The results of this study demonstrate that the combination of PNF and Str significantly improves skeletal muscle fibrosis in rats, with the underlying mechanism potentially associated with modulation of the aberrantly expressed TGF-β1/Smad signaling pathway. The combination of PNF and Str demonstrated significant therapeutic effects on fibrotic muscle tissue, including (1) marked increase in muscle tissue length; (2) substantial improvements in functional parameters (joint ROM, hindlimb grip strength, gastrocnemius twitch and tetanic contraction forces); (3) reduction of the expression of fibrosis markers (collagen I, collagen III, CSA，CVF%); and (4) effective regulation of key TGF-β1/Smad pathway components—upregulating TGF-β1, Smad2, Smad3, and α-SMA and downregulating Smad7 and IFN-γ. In contrast, the Apu + Str intervention failed to demonstrate significant improvements in muscle function, gastrocnemius length, or several key fibrosis indicators, suggesting limited therapeutic efficacy for early-stage skeletal muscle fibrosis. Notably, the PNF + Str intervention showed markedly superior treatment outcomes than the Apu + Str intervention. While the measurement of p-Smad2/3 would provide more direct evidence of pathway activity, the coordinated downregulation of total Smad2/3 and the upstream regulator TGF-β1, coupled with the upregulation of Smad7, provides compelling indirect evidence supporting the conclusion of pathway inhibition.

The joint ROM and hindlimb grip strength serve as reliable indicators of hindlimb muscular function and have notable diagnostic and prognostic value. The single contraction force represents the maximal force generated during an isolated muscle contraction, while the tetanic contraction force reflects the sustained force output during continuous muscle activation. As established in previous research [22], quantitative assessments of these contractile properties can provide crucial information about muscular functional status during fibrotic progression, contributing substantially to our understanding of the pathogenesis of skeletal muscle fibrosis and serving as vital parameters for evaluating therapeutic outcomes. The study by Wang et al. [23] investigated the effects of different exercise interventions on grip strength, knee-extension strength, and skeletal muscle index in patients with sarcopenia. Their findings demonstrated that improving these muscle-function parameters can effectively enhance skeletal muscle health status. In this study, both imm + PNF + Str and Apu + Str interventions significantly improved multiple functional parameters in rat hindlimbs, including joint ROM, grip strength, single contraction force and tetanic contraction force of the gastrocnemius muscle, and the muscle length and weight. Notably, imm + PNF + Str demonstrated superior efficacy in increasing the gastrocnemius muscle length than imm + Apu + Str. These findings suggest that the flat-blade design of PNF instruments enables more effective release of fascial contractures, thereby alleviating muscle fibrosis and significantly enhancing muscular extensibility.

Masson's trichrome staining, which enables quantitative assessment of collagen fiber content through a distinct color contrast, is an established method for evaluating the severity of muscle damage in myodystrophy, rhabdomyolysis, and related disorders. In the current study, the imm + PNF + Str and imm + Apu + Str interventions demonstrated significant therapeutic effects, including reduction in muscle fiber diameter, decreased intermuscular spacing, improved fiber alignment, and markedly improved CSA, reduced CVF%. These morphological improvements collectively indicate substantial alleviation of skeletal muscle fibrosis, with imm + PNF + Str showing particularly superior therapeutic outcomes than imm + Apu + Str.

Our study revealed an important finding: although there were no differences in cross-sectional area or muscle weight between the PNF and Apu groups, the PNF group demonstrated significant advantages in promoting functional recovery. This superior recovery was not attributable to muscle fiber hypertrophy or increased muscle mass. Instead, we propose that the exceptional efficacy of PNF primarily stems from its more effective reversal of fibrosis and consequent improvement in muscle functional quality and/or the microenvironment. When skeletal muscle becomes fibrotic, force transmission within the muscle is compromised. PNF appears to mitigate this pathological force dissipation more effectively, thereby allowing a greater proportion of the force generated during muscle contraction to be translated into practical external work.

In skeletal muscle fibrosis, collagen I primarily contributes to pathological progression by increasing ECM stiffness and rigidity, thereby restricting cellular functionality and regenerative capacity. In contrast, collagen III provides elastic support during the early fibrotic stages [24], helping maintain ECM flexibility and resilience. The deposition patterns of collagen I and III undergo dynamic changes during the progression of skeletal muscle fibrosis: in early-stage fibrosis, collagen III predominates as the primary ECM component, while fibrotic progression leads to gradual dominance of collagen I deposition [25]. In this study, the mean fluorescence intensities of collagen I and III in the gastrocnemius muscle were analyzed by immunofluorescence analyses. Both collagen I and III showed significant elevation post-immunisation. Notably, the expression level of collagen III in the Model group exhibited a pronounced disparity in comparison with the Control group, suggesting that the disease modeled in this experiment represents an early fibrotic stage. Given the observed alterations in collagen III expression, future experiments could extend the modeling duration to monitor disease progression dynamics. Both imm + PNF + Str and imm + Apu + Str groups showed significantly decreased expression levels of collagen I and III, with the collagen III level exhibiting the most marked reduction. Although the Apu + Str group showed a decline in collagen III expression, the difference lacked statistical significance. These results further indicate that imm + PNF + Str provides a more pronounced therapeutic effect than imm + Apu + Str in early-stage skeletal muscle fibrosis.

**Fig. 8:**
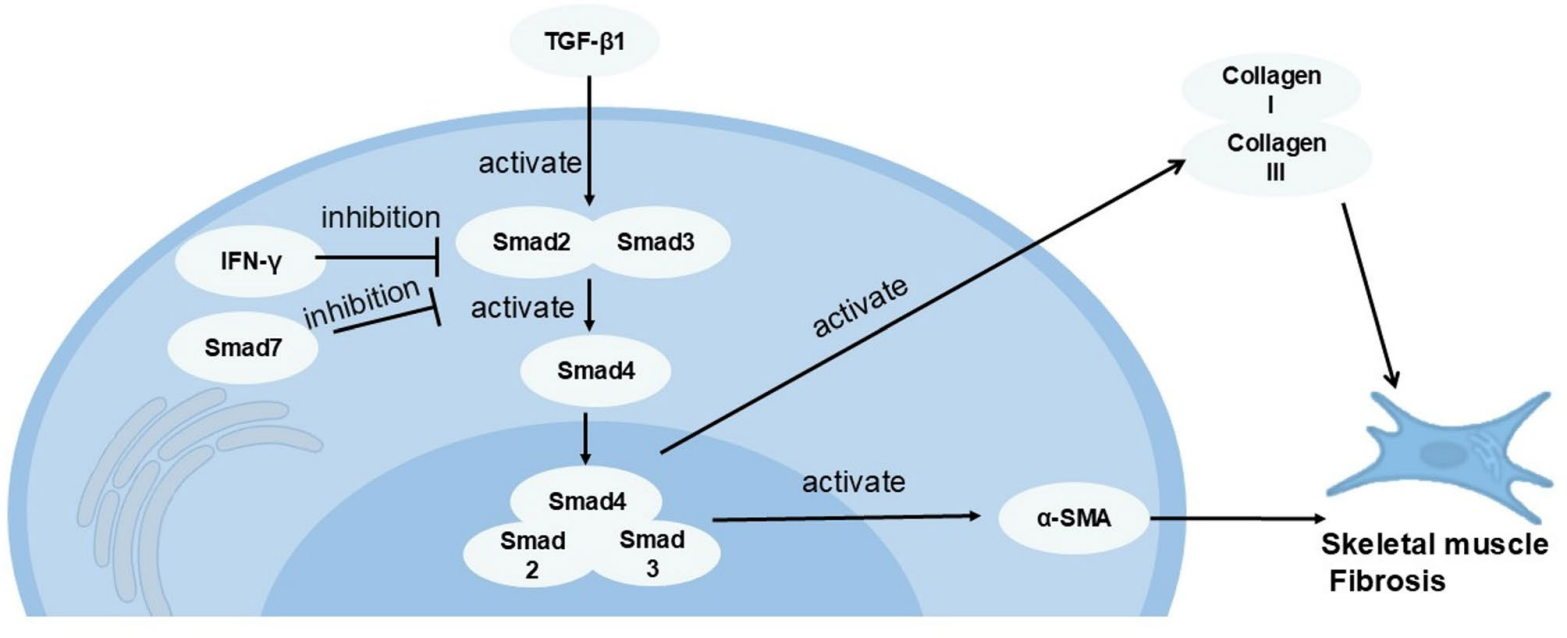
The pathogenic mechanism involving the activation of the TGF-β1/Smad signaling pathway and its role in skeletal muscle fibrosis

The TGF-β1/Smad signaling pathway serves as a pro-fibrotic pathway that plays important roles in skeletal muscle fibrosis. Within this pathway, TGF-β1 [26] acts as a potent pro-fibrotic cytokine. TGF-β1 [27] activates Smad2/3 proteins, which then form a protein complex with Smad4 and translocate into the nucleus to regulate gene expression. This process promotes the expression of α-SMA and stimulates excessive synthesis of ECM components, ultimately leading to skeletal muscle fibrosis. Smad7 serves as a crucial negative regulator of the TGF-β1 signaling pathway [28], which functions by recognizing and binding to TGF-β1 receptors to form stable complexes, thereby hindering the access of Smad2 and Smad3 to these receptors. Additionally, IFN-γ can suppress the TGF-β1 signaling pathway through STAT1 [29], either by inhibiting the phosphorylation of Smad2/Smad3 or by upregulating Smad7 expression (as shown in Fig. 8). In this study, we analyzed the mRNA and protein expression levels of TGF-β1, Smad2, Smad3, and α-SMA in the gastrocnemius muscle, which were significantly elevated after 4 weeks of modeling, while the mRNA and protein levels of Smad7 and IFN-γ were markedly reduced, suggesting that aberrant activation of the TGF-β1/Smad signaling pathway may contribute to skeletal muscle fibrosis. In the assessment of interventions, both imm + PNF + Str and imm + Apu + Str treatments significantly decreased the mRNA and protein expression of TGF-β1, Smad2, Smad3, and α-SMA, while upregulating Smad7 and IFN-γ levels, indicating attenuated skeletal muscle fibrosis post-intervention. Notably, imm + PNF + Str demonstrated more pronounced improvements than imm + Apu + Str.

While our study provides compelling evidence for the involvement of the TGF-β1/Smad pathway in the anti-fibrotic effects of PNF combined with Str, we acknowledge that skeletal muscle fibrosis is a complex process orchestrated by multiple interconnected signaling networks. These include downstream effectors such as connective tissue growth factor (CTGF) [30], which amplifies the pro-fibrotic response; the imbalance between matrix metalloproteinases (MMPs) and their inhibitors (TIMPs) [31], which governs ECM remodeling; and key inflammatory cytokines (e.g., IL-6 [32], TNF-α [31]) that initiate the fibrotic cascade. Furthermore, the pathway is subject to intricate upstream regulation, as exemplified by a recent finding that miR-27b-3p [33] attenuates muscle fibrosis by directly targeting TGF-β receptor I (TGF-βR1) and suppressing Smad3 phosphorylation. Therefore, although our findings robustly highlight the TGF-β1/Smad axis as a primary target, they also lay the groundwork for future studies to explore its crosstalk with these broader regulatory networks for a more comprehensive mechanistic understanding.

## Conclusion

In conclusion, PNF combined with Str intervention effectively mitigated skeletal muscle fibrosis, likely through its regulatory effects on the TGF-β1/Smad signaling pathway. The pathological changes observed in this study indicate that the disease model corresponds to an early-stage fibrotic condition. On the basis of these findings, future experiments should consider either prolonging the modeling duration or optimizing modeling techniques. Such modifications would enable more comprehensive observation and evaluation of different interventions' impact on fibrosis progression, thereby providing a stronger foundation for developing effective therapeutic strategies against skeletal muscle fibrosis.However, these findings require further validation in clinical studies.

## Limitations

While this study demonstrates the superior therapeutic effect of combining PNF with Str, the experimental design lacked groups treated with PNF or Str alone. Therefore, we cannot statistically distinguish between an additive or synergistic interaction using methods like two-way ANOVA. Future studies incorporating the necessary factorial design are needed to definitively elucidate their interaction.A key limitation is that we assessed total protein levels of key TGF-β1/Smad pathway components (Smad2, Smad3, Smad7) but not their phosphorylation status (p-Smad2/3), which is the direct marker of pathway activation. Despite this, the coordinated regulation of these molecules and the downstream effector α-SMA provides strong indirect evidence that the anti-fibrotic effect of PNF + Str involves suppression of the TGF-β1/Smad pathway. Direct measurement of p-Smad2/3 will be a priority in future work to solidify this mechanistic.

## Abbreviations

PNF: Acupotomy Percutaneous Needle Fasciotomy
imm: Immobilization
Apu: Acupuncture
Str: Stretching
ROM: Range of motion
ECM: Extracellular matrix
TGF-β1: Transforming growth factor beta-1
IFN-γ: Interferon gamma
α-SMA: Alpha smooth muscle actin
